# Supplementary material for: Characterization of covalent inhibitors that disrupt the interaction between the tandem SH2 domains of SYK and FCER1G phospho-ITAM
Source: PLoS One. 2024 Feb 15;19(2):e0293548. doi: 10.1371/journal.pone.0293548 (PMC10868801; doi:10.1371/journal.pone.0293548)
Supplement: S1 Table — Chemical structures of the initial hit from uHTS (39) and analogues purchased based on this hit compound. TR-FRET data are reported as the IC50 ± standard deviation (n = 3). DSF data are reported as ΔTm ± standard deviation (n = 3). a TR-FRET data from initial uHTS. NT = Not tested. (PDF) [file pone.0293548.s001.pdf]

UNC#	Structure	TR-FRET (IC_{50}) μM	DSF (ΔT_m)
**39**	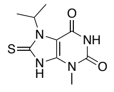	27.17[a]	NT
**43**	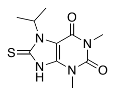	1.9 ± 0.20	-16.6 ± 0.3
**42**	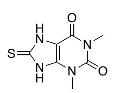	3.0 ± 1.7	-6.7 ± 0.4
**45**	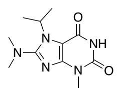	> 100	NT
**46**	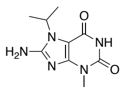	> 100	NT
**47**	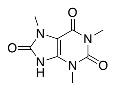	>100	NT
**48**	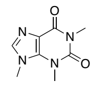	>100	NT
**49**	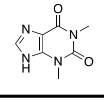	>100	NT
**50**	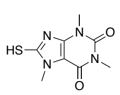	3.57 ± 0.20	NT
**51**	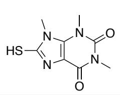	2.93 ± 0.16	NT

UNC#	Structure	TR-FRET (IC_{50}) μM	DSF (ΔT_m)
**52**	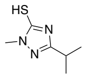	5.41± 0.06	NT
**53**	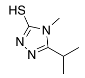	4.94± 0.23	NT
**54**	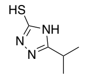	12.52± 0.57	NT
**55**	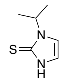	9.7 ± 4.3	NT
**56**	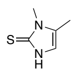	15 ± 3.5	NT
**57**	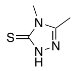	>100	NT
**58**	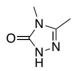	>100	NT
**59**	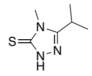	7.7 ± 1.0	NT
