# Supplementary figures and images for: Characterization of covalent inhibitors that disrupt the interaction between the tandem SH2 domains of SYK and FCER1G phospho-ITAM
Source: PLoS One. 2024 Feb 15;19(2):e0293548. doi: 10.1371/journal.pone.0293548 (PMC10868801; doi:10.1371/journal.pone.0293548)

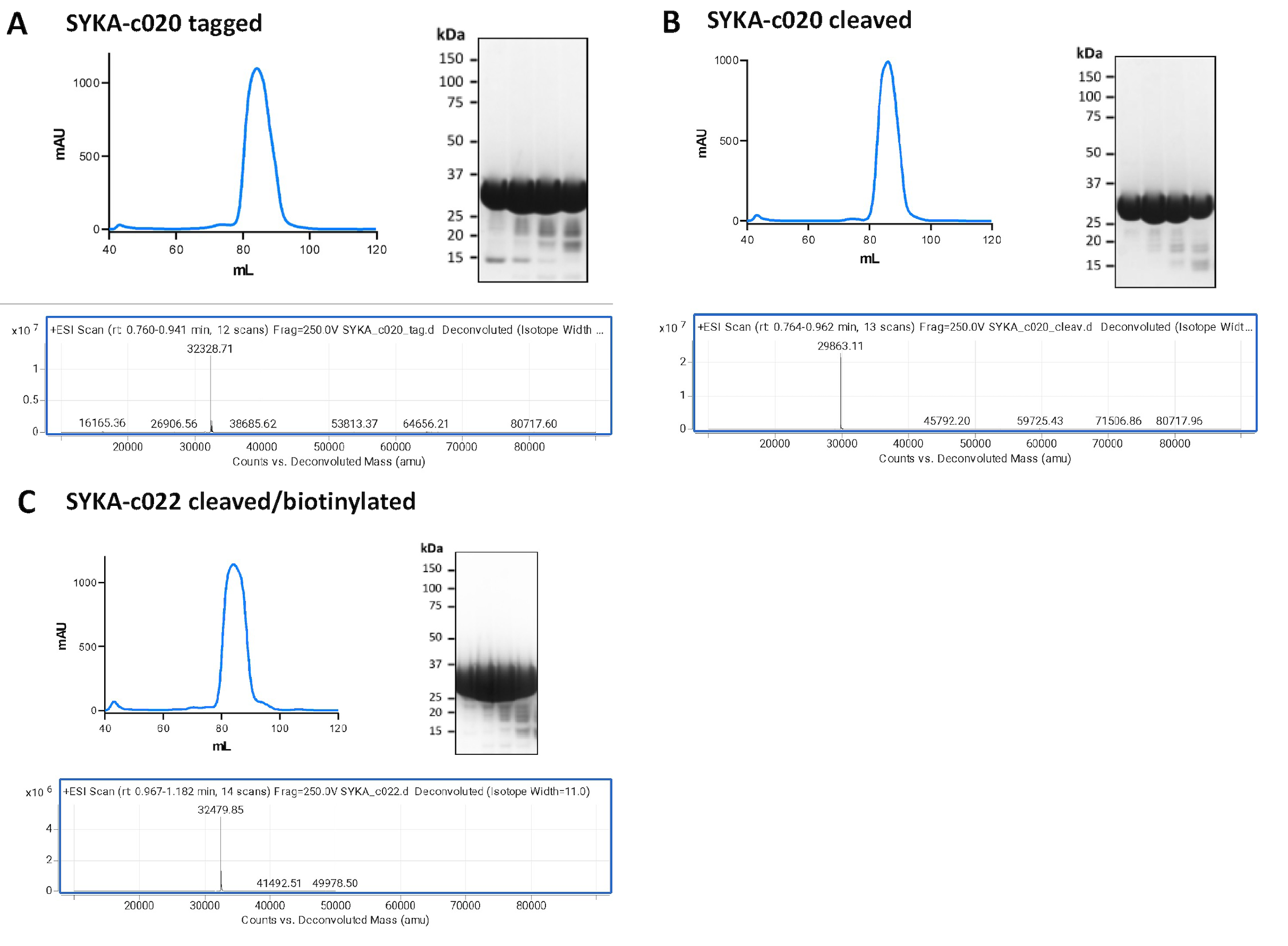

Supplement: S1 Fig — (A) SYKA-c020 His tagged, (B) SYKA-c020 His tag cleaved, and (C) SYKA-c020 His tag cleaved and biotinylated. (TIFF) [file pone.0293548.s002.tiff]

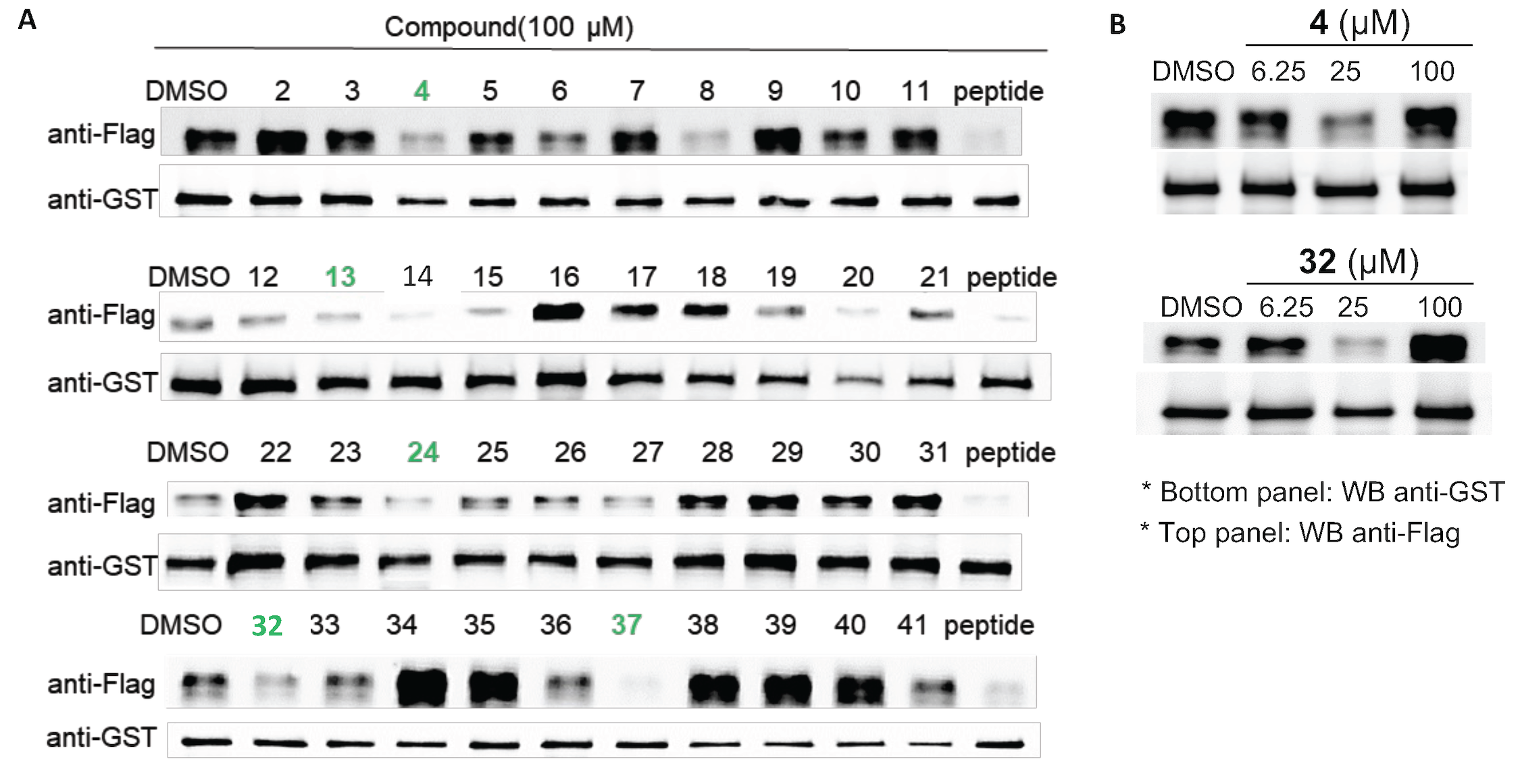

Supplement: S2 Fig — (A) Compounds 4, 13, 24, 32, and 37, 4, were able to inhibit the interaction between SYK-GST and FCER1G-Flag in a pulldown assay at a single concentration (100 μM). Second biological replicate displayed. (B) Dose-response GST-PD of 4 and 32. Unmodified gels corresponding with panels (A) and (B) are included in S7 Fig. (TIFF) [file pone.0293548.s003.tiff]

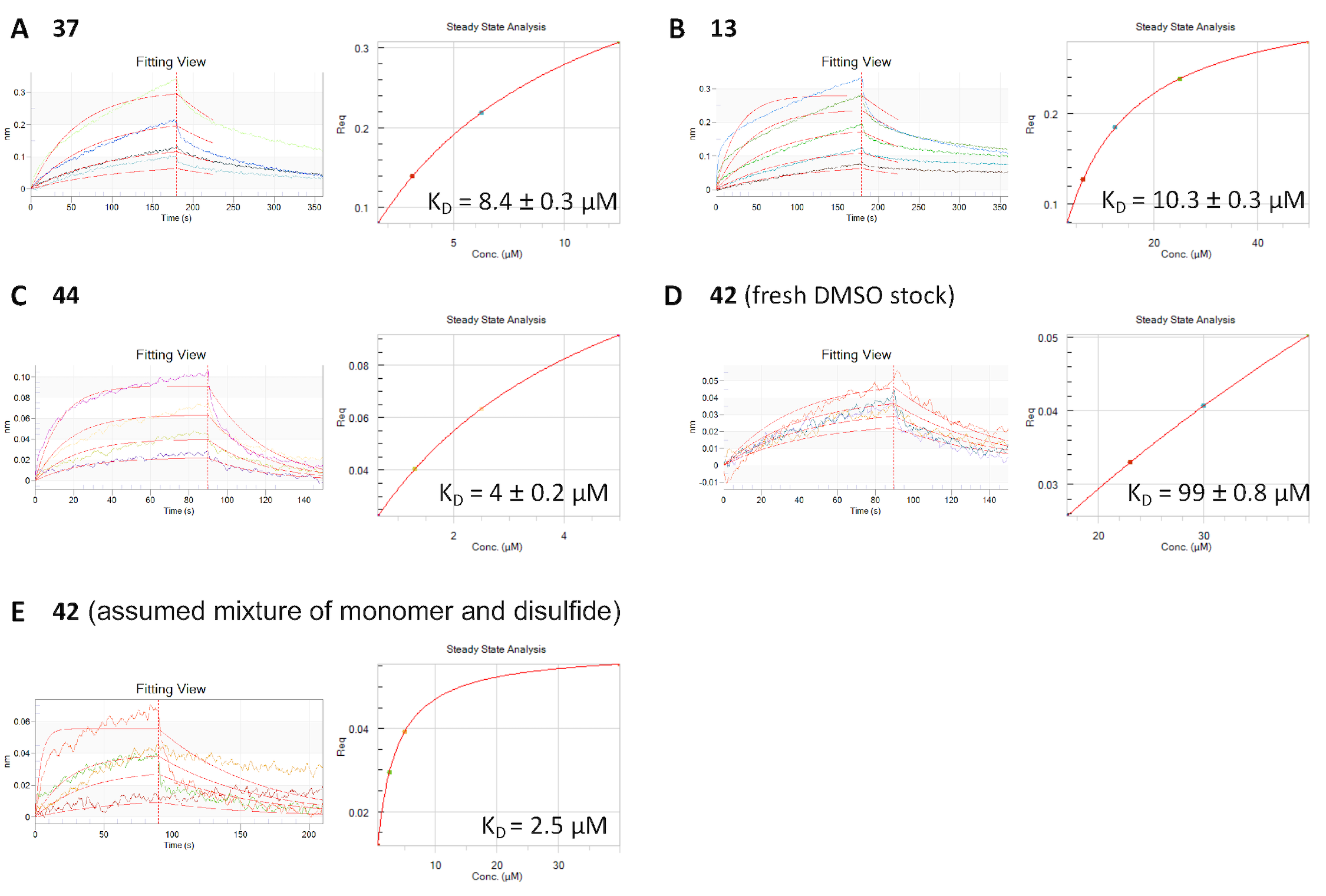

Supplement: S3 Fig — (A) 37, (B) 13, (C) 44, (D) 42 (fresh DMSO stock–monomer compound), (E) 42 (assumed mixture of monomer and disulfide) BLI data. (TIFF) [file pone.0293548.s004.tiff]

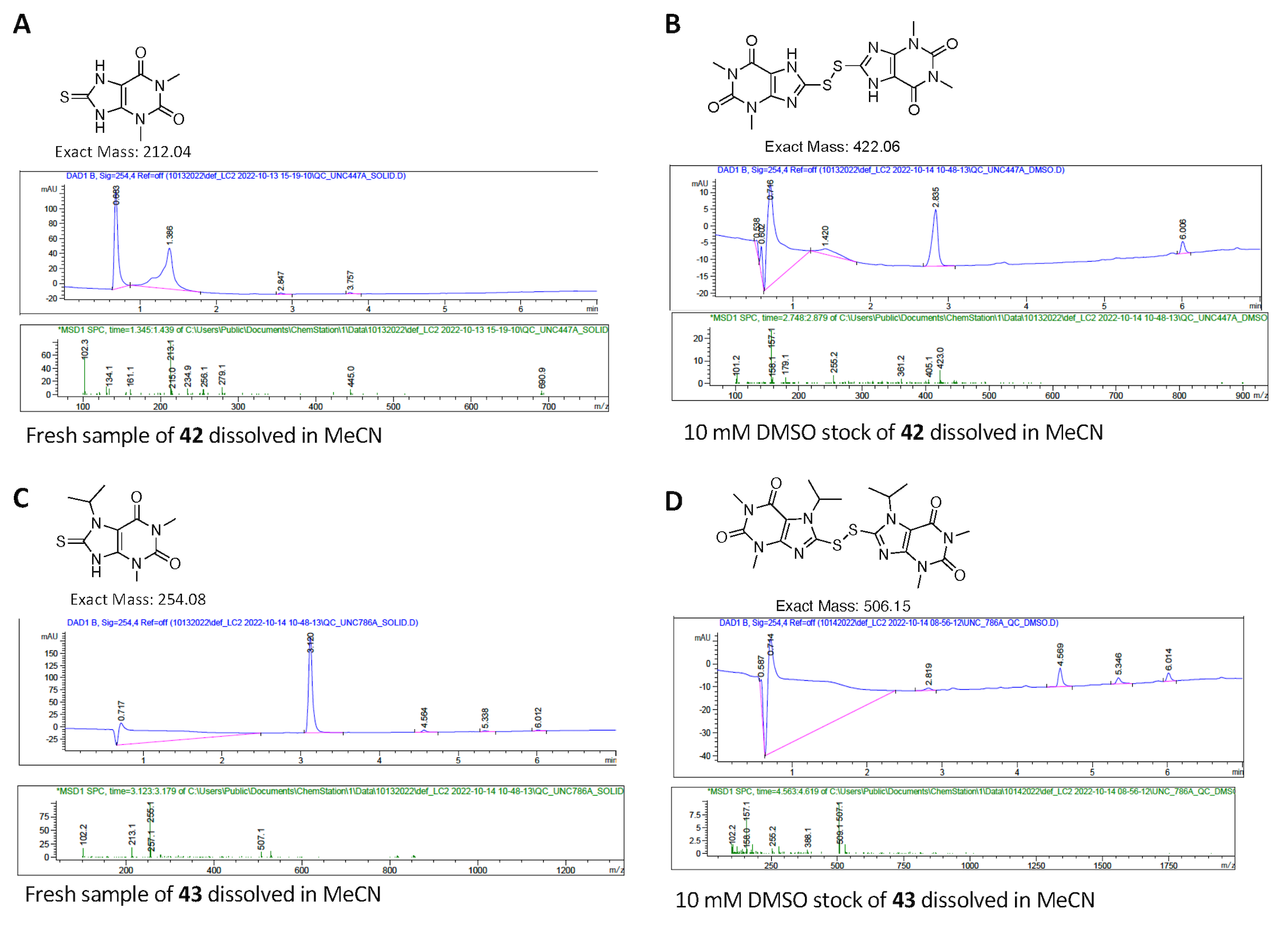

Supplement: S4 Fig — (A) LCMS analysis of 42 as a fresh solution in MeCN indicates the monomer is present. LCMS Calculated for [M+H]+ C7H9N4O2S: 213.04; observed: 213.1 [M+H]+. (B) LCMS analysis of 42 10 mM stock solution in MeCN indicates the disulfide dimer is present. LCMS Calculated for [M+H]+ C14H15N8O4S2: 423.06; observed: 423.0 [M+H]+. (C) LCMS analysis of 43 as a fresh solution in MeCN indicates the monomer is present. LCMS Calculated for [M+H]+ C10H15N4O2S: 255.08; observed: 255.1 [M+H]+. (D) LCMS analysis of 43 10 mM stock solution in MeCN indicates the disulfide dimer is present. LCMS Calculated for [M+H]+ C20H27N8O4S2: 507.15; observed: 507.1 [M+H]+. (TIFF) [file pone.0293548.s005.tiff]

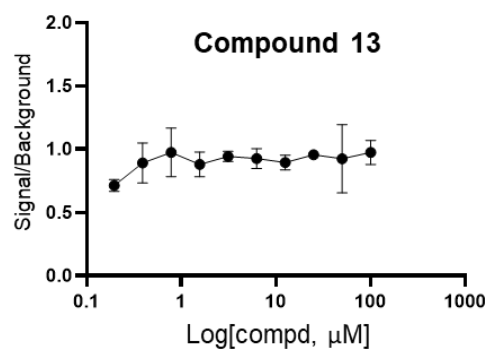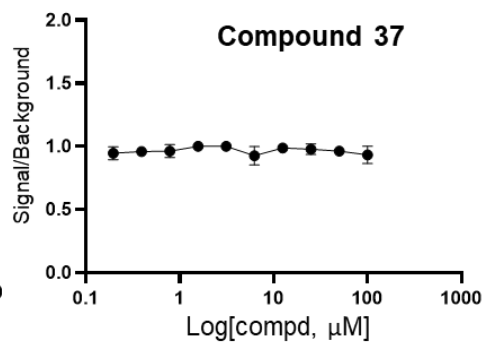

Supplement: S5 Fig — (PDF) [file pone.0293548.s006.pdf]

## A SYK

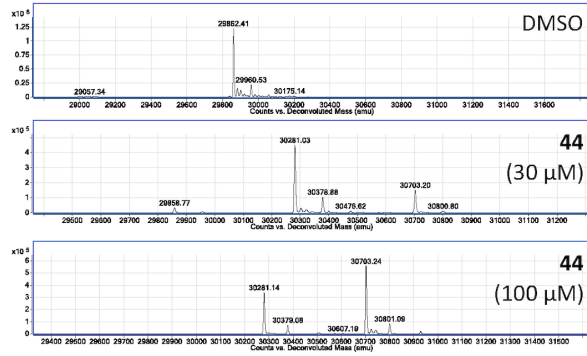

## B SHIP1

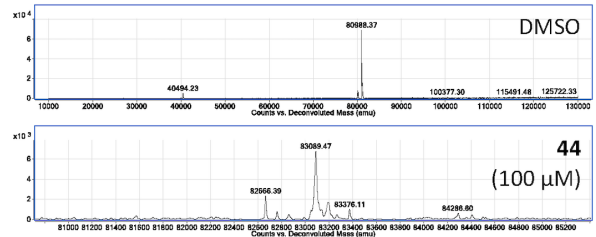

## C TBXT

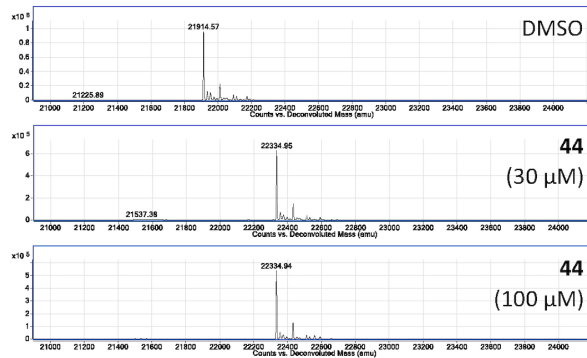

## D MSN

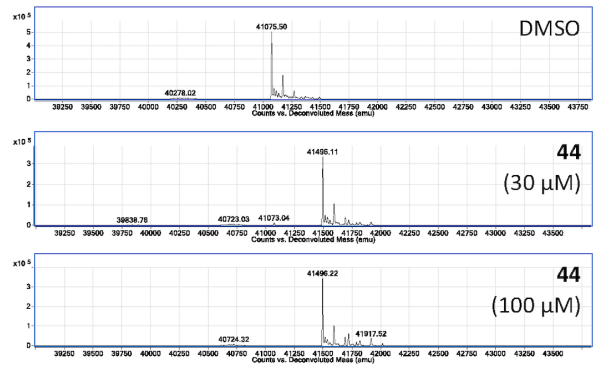

Supplement: S6 Fig — (A) SYK, (B) SHIP1, (C) TBXT, (D) MSN. (PDF) [file pone.0293548.s007.pdf]
