# Supplementary material for: Characterization of covalent inhibitors that disrupt the interaction between the tandem SH2 domains of SYK and FCER1G phospho-ITAM
Source: PLoS One. 2024 Feb 15;19(2):e0293548. doi: 10.1371/journal.pone.0293548 (PMC10868801; doi:10.1371/journal.pone.0293548)
Supplement: S7 Fig — These images correspond with data included in Table 1 as well as blots in Figs 4A–4C, 6H, and S2A, S2B Fig. (PDF) [file pone.0293548.s008.pdf]

**Figure 4A.**

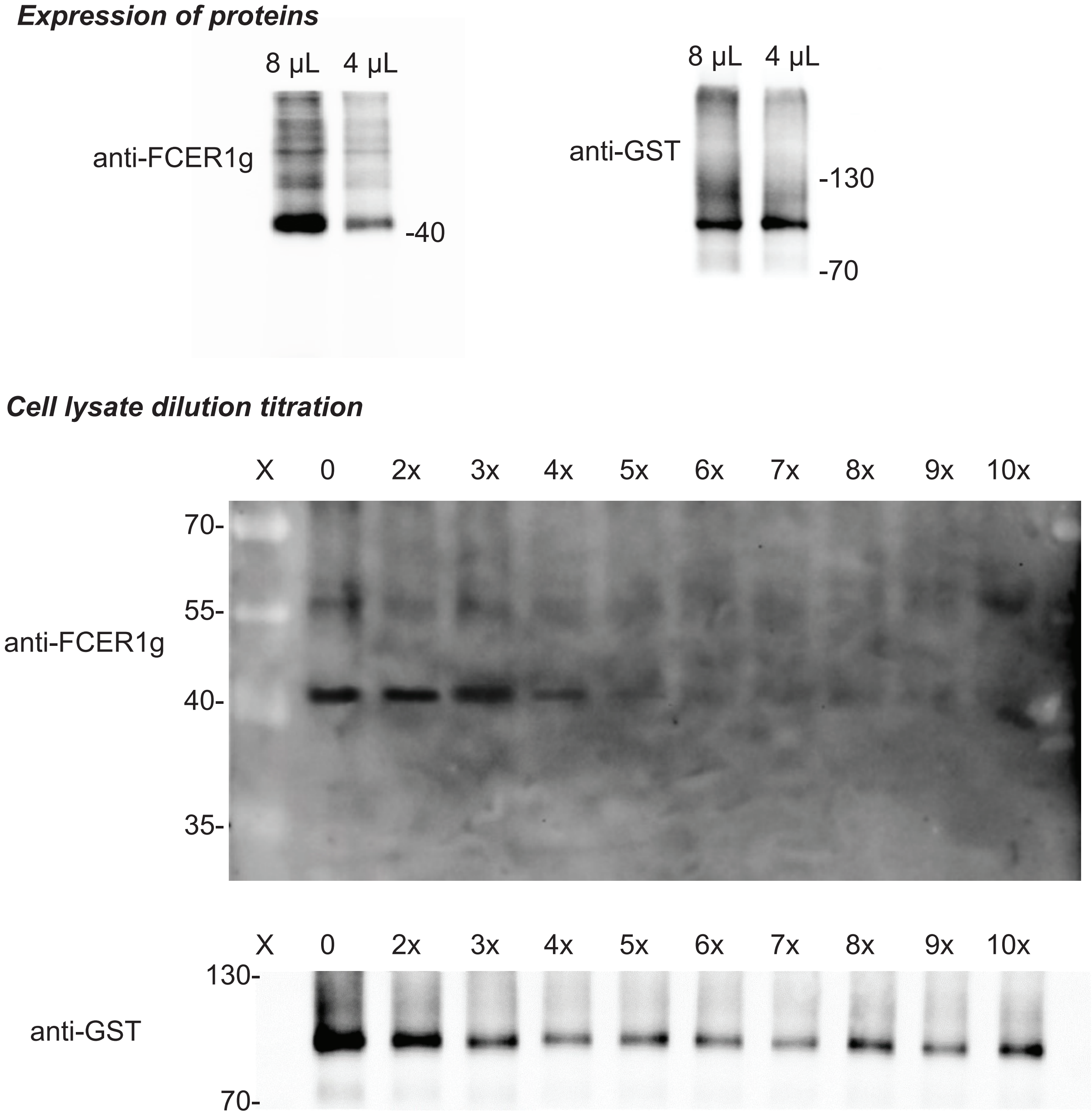
Expression of proteins
8 µL
4 µL
anti-FCER1g
-40
8 µL
4 µL
anti-GST
-130
-70
Cell lysate dilution titration
X
0
2x
3x
4x
5x
6x
7x
8x
9x
10x
70-
55-
anti-FCER1g
40-
35-
X
0
2x
3x
4x
5x
6x
7x
8x
9x
10x
130-
anti-GST
70-

**Figure 4B**

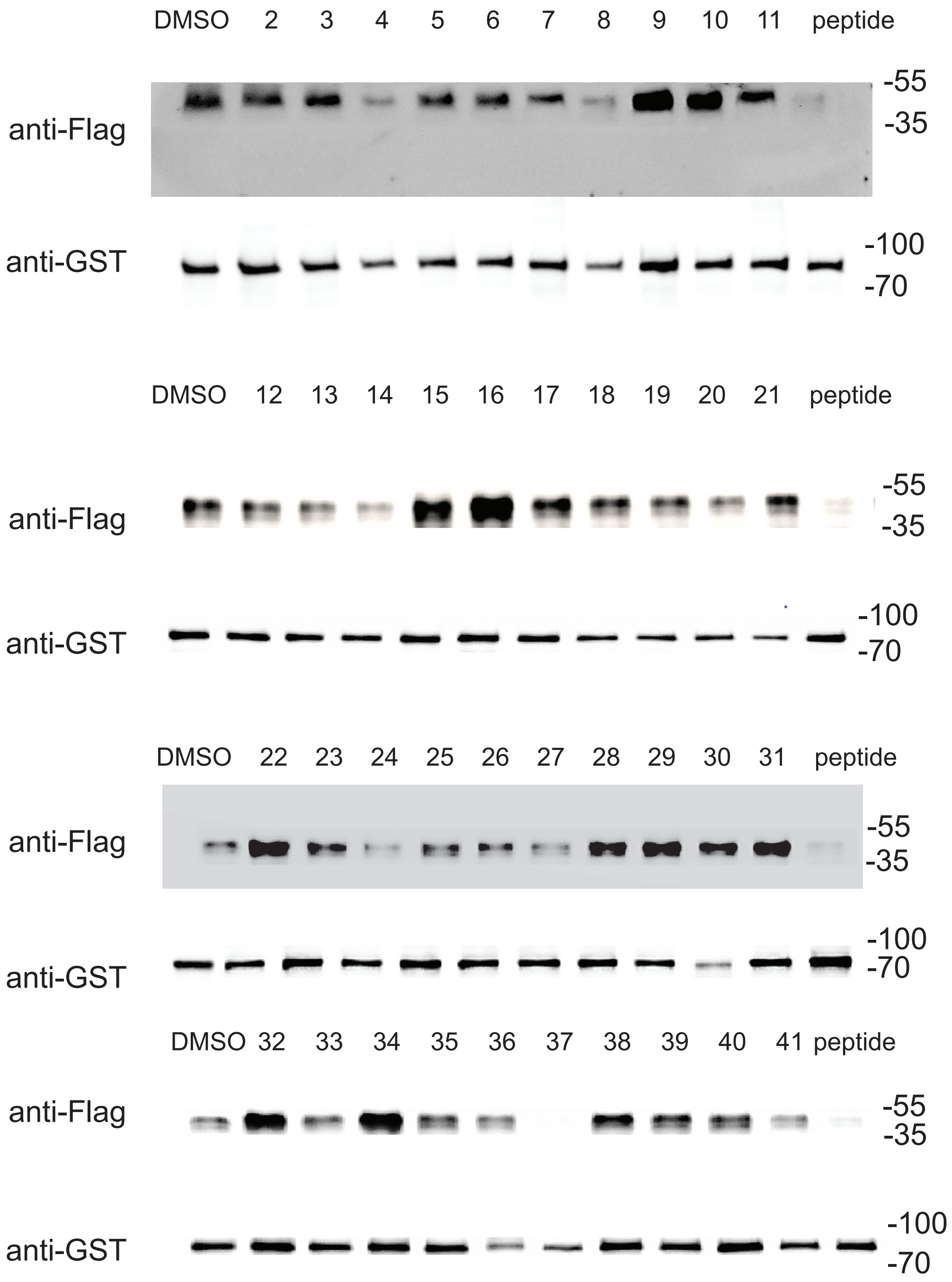
DMSO 2 3 4 5 6 7 8 9 10 11 peptide
anti-Flag
-55
-35
anti-GST
-100
-70
DMSO 12 13 14 15 16 17 18 19 20 21 peptide
anti-Flag
-55
-35
anti-GST
-100
-70
DMSO 22 23 24 25 26 27 28 29 30 31 peptide
anti-Flag
-55
-35
anti-GST
-100
-70
DMSO 32 33 34 35 36 37 38 39 40 41 peptide
anti-Flag
-55
-35
anti-GST
-100
-70

# Figure 4C

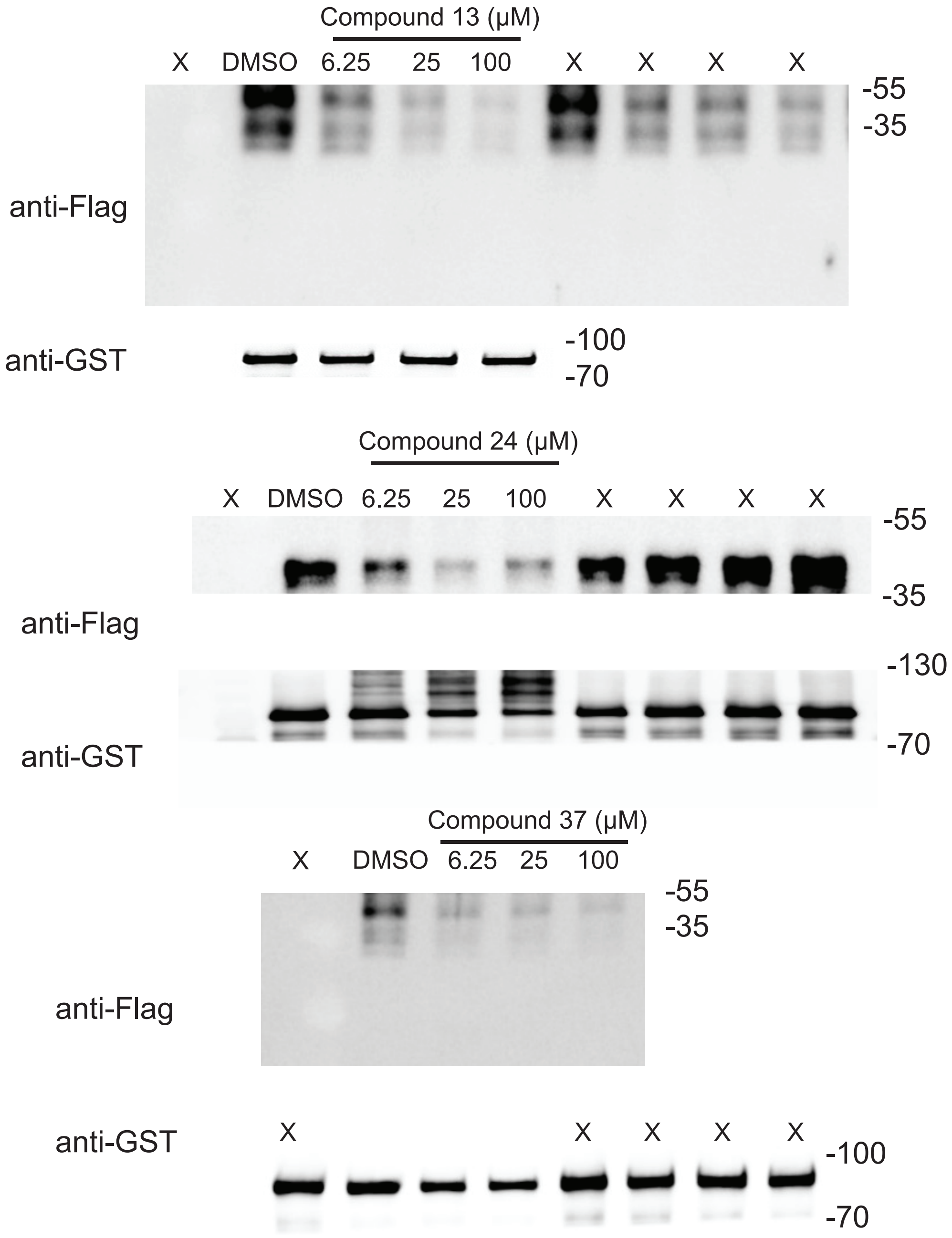

Compound 13 (μM)
X DMSO 6.25 25 100 X X X X
-55
-35
anti-Flag
anti-GST
-100
-70
Compound 24 (μM)
X DMSO 6.25 25 100 X X X X
-55
-35
anti-Flag
-130
anti-GST
-70
Compound 37 (μM)
X DMSO 6.25 25 100
-55
-35
anti-Flag
anti-GST
X X X X X
-100
-70

**Figure 6H**

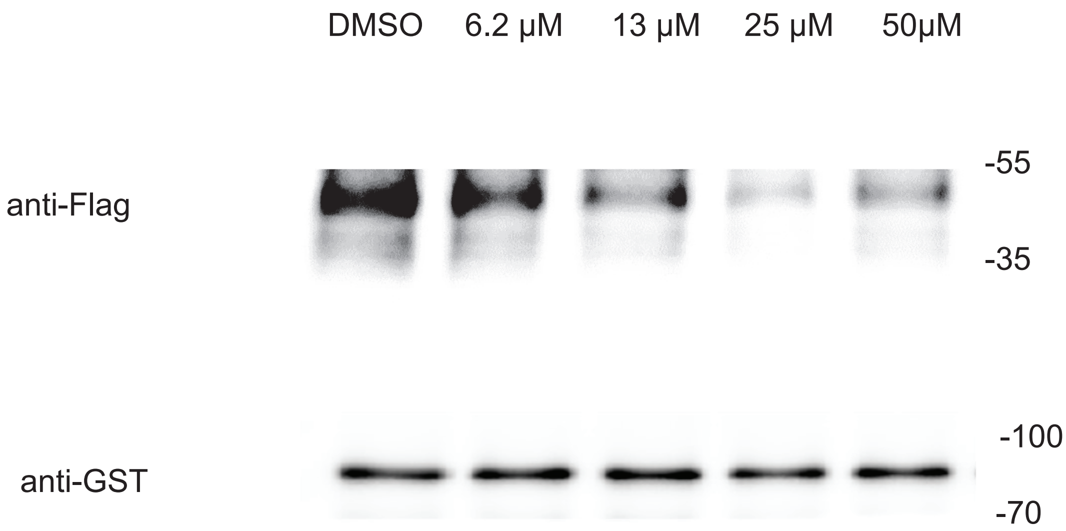

## Figure SI 2A

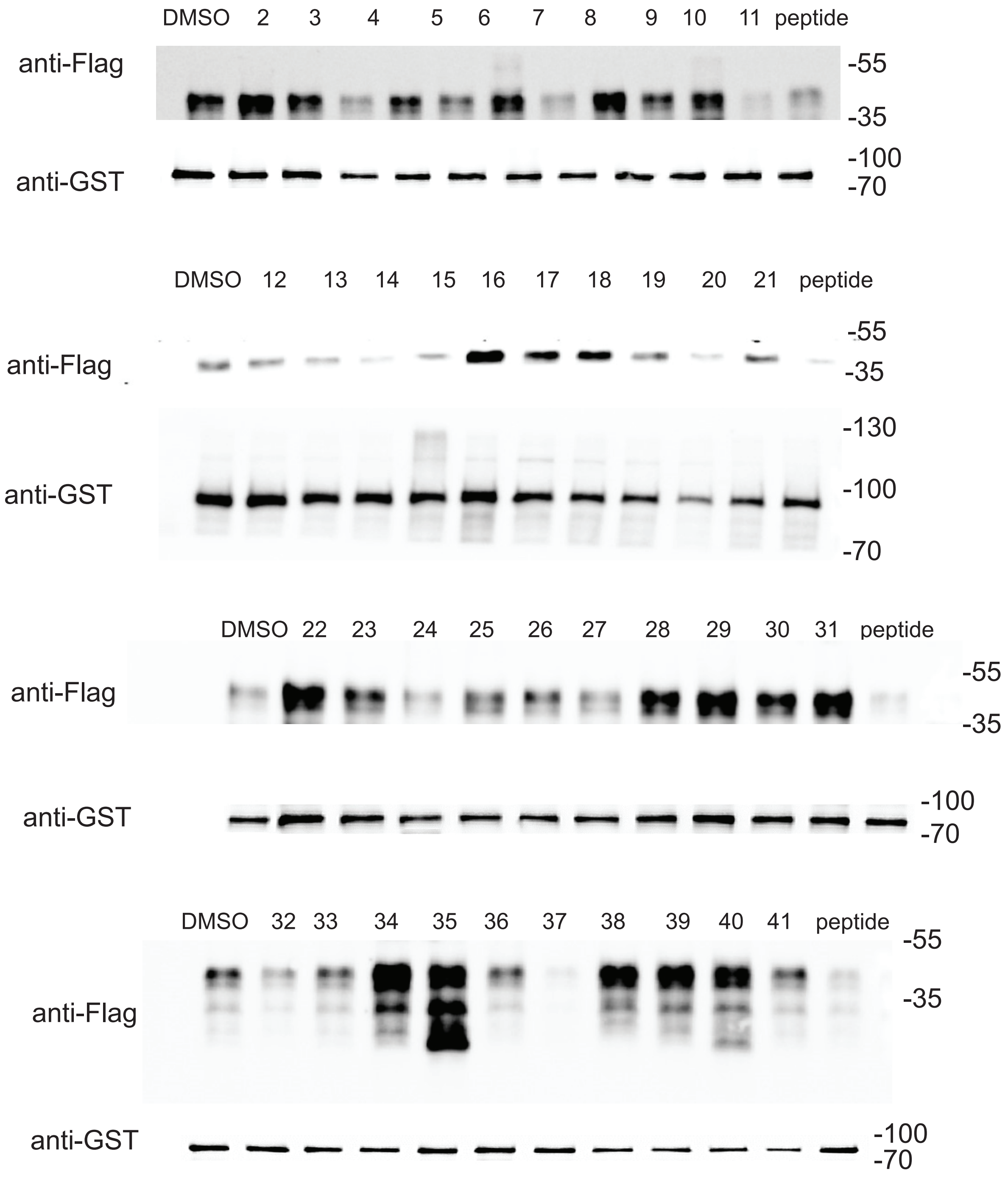
DMSO 2 3 4 5 6 7 8 9 10 11 peptide
anti-Flag
-55
-35
anti-GST
-100
-70
DMSO 12 13 14 15 16 17 18 19 20 21 peptide
anti-Flag
-55
-35
anti-GST
-130
-100
-70
DMSO 22 23 24 25 26 27 28 29 30 31 peptide
anti-Flag
-55
-35
anti-GST
-100
-70
DMSO 32 33 34 35 36 37 38 39 40 41 peptide
anti-Flag
-55
-35
anti-GST
-100
-70

**Figure SI 2B**

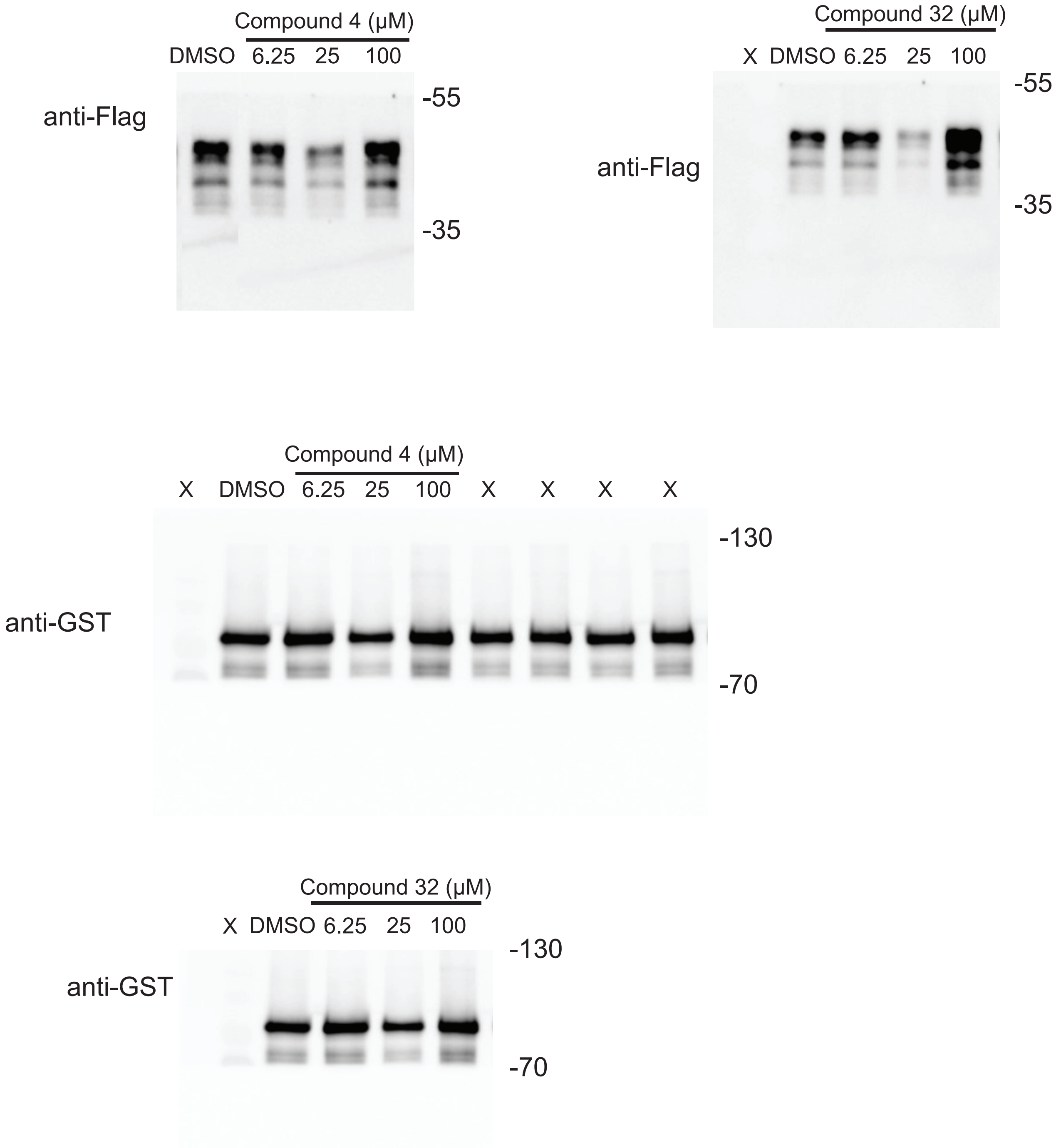

Compound 4 (μM)
DMSO 6.25 25 100
anti-Flag
-55
-35
Compound 32 (μM)
X DMSO 6.25 25 100
anti-Flag
-55
-35
Compound 4 (μM)
X DMSO 6.25 25 100 X X X X
anti-GST
-130
-70
Compound 32 (μM)
X DMSO 6.25 25 100
anti-GST
-130
-70
