# Supplementary material for: Characterization of covalent inhibitors that disrupt the interaction between the tandem SH2 domains of SYK and FCER1G phospho-ITAM
Source: PLoS One. 2024 Feb 15;19(2):e0293548. doi: 10.1371/journal.pone.0293548 (PMC10868801; doi:10.1371/journal.pone.0293548)

^{1}H NMR of 8,8'-disulfanediylbis(1,3-dimethyl-3,7-dihydro-1*H*-purine-2,6-dione) (44)

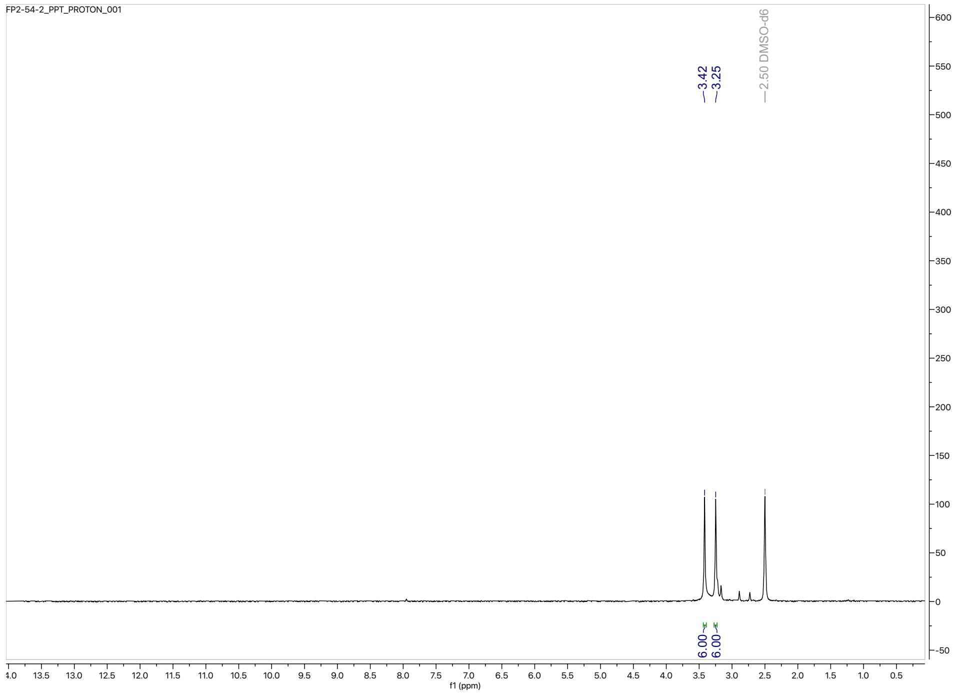

LCMS of 8,8'-disulfanediylbis(1,3-dimethyl-3,7-dihydro-1*H*-purine-2,6-dione) (44)

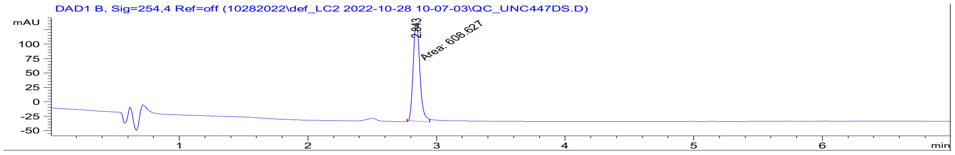

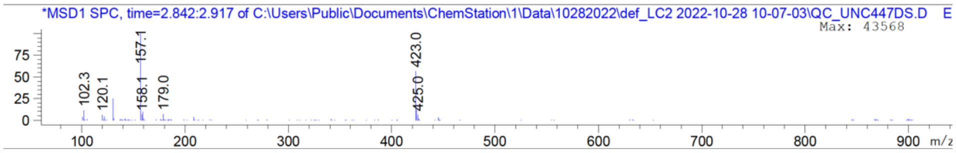

Supplement: S8 Fig — 1H NMR and LCMS spectra that confirm identity and purity of compound 44. (PDF) [file pone.0293548.s009.pdf]
