# Supplementary material for: Characterization of covalent inhibitors that disrupt the interaction between the tandem SH2 domains of SYK and FCER1G phospho-ITAM
Source: PLoS One. 2024 Feb 15;19(2):e0293548. doi: 10.1371/journal.pone.0293548 (PMC10868801; doi:10.1371/journal.pone.0293548)
Supplement: Attachment — Submitted filename: Response to Reviewers.pdf [file pone.0293548.s010.pdf]

Journal: PLoS ONE
Manuscript ID: PONE-D-23-24596
Title: "Characterization of covalent inhibitors that disrupt the interaction between the tandem SH2 domains of SYK and FCER1G phospho-ITAM"
Author list: Bashore, F. M.; Katis, V. L.; Du, Y.; Sikdar, A.; Wang, D.; Bradshaw, W. J.; Rygiel, K. A.; Leisner, T. M.; Chalk, R.; Mishra, S.; Williams, C. A.; Gileadi, O.; Brennan, P. E.; Wiley, J. C.; Gockley, J.; Cary, G. A.; Carter, G. W.; Young, J. E.; Pearce, K. H.; Fu, H.; the Emory-Sage-SGC TREAT-AD Center; Axtman, A. D.

Journal Requirements:

1. Please ensure that your manuscript meets PLOS ONE's style requirements, including those for file naming. The PLOS ONE style templates can be found at https://journals.plos.org/plosone/s/file?id=wjVg/PLOSOne_formatting_sample_main_body.pdf and https://journals.plos.org/plosone/s/file?id=ba62/PLOSOne_formatting_sample_title_authors_affiliations.pdf
   Author reply: We have made amendments to the manuscript and supporting information so that both documents adhere to PLOS ONE's style requirements. This has included updating the References section, which is described in more detail below.

2. We note that the grant information you provided in the 'Funding Information' and 'Financial Disclosure' sections do not match.

   When you resubmit, please ensure that you provide the correct grant numbers for the awards you received for your study in the 'Funding Information' section.
   Author reply: The Funding Information section as entered in the online submission system is correct. The Funding Information statement, as included in the cover letter, has been updated to match this information.

3. Please expand the acronym "NIA" (as indicated in your financial disclosure) so that it states the name of your funders in full.

   This information should be included in your cover letter; we will change the online submission form on your behalf.
   Author reply: The acronym "NIA" has been expanded and this information is also listed in our cover letter.

4. Thank you for stating the following in the Acknowledgments Section of your manuscript:
   "The authors thank Drs. Allan I. Levey, Lara M. Mangravite, Haian Fu, Opher Gileadi, Aled Edwards, Frank Longo, Gregory W. Carter, Tim M. Willson, and Stephen V. Frye for insightful discussions and suggestions during the development of these resources. The Target Enablement to Accelerate Therapy Development for Alzheimer's Disease (TREAT-AD) Consortium was established by the National Institute on Aging (NIA). The research reported in this manuscript was led by the Emory-Sage-SGC TREAT-AD center and supported by grant U54AG065187 from the NIA. The Structural Genomics Consortium (SGC) is a registered charity (number 1097737) that receives funds from Bayer AG, Boehringer Ingelheim, the Canada Foundation for Innovation, Eshelman Institute for Innovation, Genentech, Genome Canada through Ontario Genomics Institute [OGI-196], EU/EFPIA/OICR/McGill/KTH/Diamond, Innovative Medicines Initiative 2 Joint Undertaking [EUbOPEN grant 875510], Janssen, Merck KGaA (aka EMD in Canada and USA), Pfizer, the São Paulo Research Foundation-FAPESP, and Takeda. The funders did not play a role in study design, data collection and interpretation, decision to publish, or manuscript preparation."

   We note that you have provided funding information that is not currently declared in your Funding Statement. However, funding information should not appear in the Acknowledgments section or other areas of your manuscript. We will only publish funding information present in the Funding Statement section of the online submission form.

   Please remove any funding-related text from the manuscript and let us know how you would like to update your Funding Statement. Currently, your Funding Statement reads as follows:
   "The research reported in this manuscript was led by the Emory-Sage-SGC TREAT-AD center and supported by grant U54AG065187 from the NIA. The Structural Genomics Consortium (SGC) is a registered charity (number 1097737) that receives funds from Bayer AG, Boehringer Ingelheim, the Canada Foundation for Innovation, Eshelman Institute for

Innovation, Genentech, Genome Canada through Ontario Genomics Institute [OGI-196], EU/EFPIA/OICR/McGill/KTH/Diamond, Innovative Medicines Initiative 2 Joint Undertaking [EUbOPEN grant 875510], Janssen, Merck KGaA (aka EMD in Canada and USA), Pfizer, the São Paulo Research Foundation-FAPESP, and Takeda. The funders did not play a role in study design, data collection and interpretation, decision to publish, or manuscript preparation."

Please include your amended statements within your cover letter; we will change the online submission form on your behalf.
Author reply: We have removed the funding information from the Acknowledgements Section of the manuscript. The amended funding statement is included in the cover letter.

5. One of the noted authors is a group or consortium [Emory-Sage-SGC TREAT-AD Center]. In addition to naming the author group, please list the individual authors and affiliations within this group in the acknowledgments section of your manuscript. Please also indicate clearly a lead author for this group along with a contact email address.
Author reply: We merged the section "members of the Emory-Sage-SGC TREAT-AD Center" with the Acknowledgements section. The authors in the group have corresponding affiliations and we have noted the group leads and their emails at the end of this information. Note: also removed Haian Fu, Opher Gileadi, and Gregory W. Carter from the Acknowledgements section as they are manuscript authors.

6. We note that you have included the phrase "data not shown" in your manuscript. Unfortunately, this does not meet our data sharing requirements. PLOS does not permit references to inaccessible data. We require that authors provide all relevant data within the paper, Supporting Information files, or in an acceptable, public repository. Please add a citation to support this phrase or upload the data that corresponds with these findings to a stable repository (such as Figshare or Dryad) and provide and URLs, DOIs, or accession numbers that may be used to access these data. Or, if the data are not a core part of the research being presented in your study, we ask that you remove the phrase that refers to these data.
Author reply: We have removed the statements "data not shown" as they are not core parts of the research being presented in the study or this phrase was used in error, as the data was actually included. These changes include:

"Compound **4** had poor solubility (25 μM, data not shown), with both **4** and **32** displaying inconsistent GST-PD data (Figure 4B and Figure S2A–B)."
Removed 'data not shown' as this information is present.

"Furthermore, we were unable to detect an effect on SYK phosphorylation in a cellular inhibition assay (data not shown), and therefore these compounds would also need to be further optimized to improve potency and ensure cell penetrance."
Removed this sentence completely, as it is not necessary. We already highlight that these compounds need further optimization and highlight their limitations.

7. PLOS ONE now requires that authors provide the original uncropped and unadjusted images underlying all blot or gel results reported in a submission's figures or Supporting Information files. This policy and the journal's other requirements for blot/gel reporting and figure preparation are described in detail at https://journals.plos.org/plosone/s/figures#loc-blot-and-gel-reporting-requirements and https://journals.plos.org/plosone/s/figures#loc-preparing-figures-from-image-files. When you submit your revised manuscript, please ensure that your figures adhere fully to these guidelines and provide the original underlying images for all blot or gel data reported in your submission. See the following link for instructions on providing the original image data: https://journals.plos.org/plosone/s/figures#loc-original-images-for-blots-and-gels.

In your cover letter, please note whether your blot/gel image data are in Supporting Information or posted at a public data repository, provide the repository URL if relevant, and provide specific details as to which raw blot/gel images, if any, are not available. Email us at plosone@plos.org if you have any questions.
Author reply: In adherence with the PLOS ONE requirements, the original uncropped and unadjusted images

underlying all blot or gel results have been added as Supporting Information (S7 Fig). This includes blots used to generate data in Table 1 as well as full, original blots or gels for images in Fig 4A–C, 6H, and S2A–2B Fig. We have noted inclusion of these unmodified gels/blots in the corresponding figure legends as well as written in our cover letter that these are not included in the Supporting Information.

8. Please review your reference list to ensure that it is complete and correct. If you have cited papers that have been retracted, please include the rationale for doing so in the manuscript text, or remove these references and replace them with relevant current references. Any changes to the reference list should be mentioned in the rebuttal letter that accompanies your revised manuscript. If you need to cite a retracted article, indicate the article's retracted status in the References list and also include a citation and full reference for the retraction notice.
   Author reply: The reference list was reviewed, and formatting updated to match the PLoS ONE style. The following changes were made to match style and ensure all references are complete: italicized journal names were changed to plain formatting, periods were removed when used to indicate the abbreviated journal name, journal names were correctly abbreviated, page numbers were added where missing, an en dash was used when page ranges were given, the entire number of the final page number was written out, and a space was inserted between the journal volume/issue and page number(s). No new references were added, and none were removed. The changes made have been mentioned in the rebuttal letter that accompanies our revised manuscript.

Response to Reviewer:

Reviewer #1:

1. Bashore et al. have screened a large (>138k) chemical library for compounds that disrupt the interaction between the SH2 domains on SYK protein tyrosine kinase and the phosphorylated ITAM domains of FCER1G. Development of such compounds has therapeutic potential in treating Alzheimer's and other diseases. They identified several compounds that efficiently disrupted the interaction when used at micromolar concentrations, and many of these effective compounds covalently attached to cysteines and other residues in the SH2 domain. The authors provide technically rigorous evidence to support their conclusions through use of a variety of state-of-the-art techniques, including FRET, bio-layer interferometry, mass spectrometry, and orthogonal-based GST pull-down assays. While I do not have any criticism of the technical aspects of the work, the authors should address two concerns:

   The primary concern for future development of these compounds as potential therapeutics is their lack of specificity to the cysteine residues in SH2 domains of Syk, since they can readily form covalent interactions with available cysteines on other proteins. Nonetheless, the approach is still interesting and future modifications may improve specificity. This lack of specificity is well documented by the authors in the Results, Discussion, and Conclusions sections, but this fact should also be prominently stated in the Abstract.
   Author reply: We thank the reviewer for highlighting the need to include a comment on the lack of specificity in the abstract of the manuscript. We have modified the last sentence of the abstract to highlight the compounds identified are not selective and should only be used with caution.

2. While the authors documented the instability of the inactive compound 42 to the active compound 44 in DMSO, they should also comment in Discussion on any other observed or potential instabilities of the active compounds that they have identified when suspended in aqueous solutions, which would be more biologically relevant.
   Author reply: We appreciate the insight offered by the reviewer. We did not note any further instabilities of compounds **42** and **44** in the presence of aqueous solutions and the transformation of **42** to **44** was only observed in 100% DMSO. The kinetic solubility of a 10 mM DMSO stock of **44** and a freshly prepared 10 mM DMSO stock of **42** was analyzed. This protocol requires that a DMSO aliquot of each is dissolved in phosphate buffered saline (PBS) solution at pH 7.4 and incubated for 24 hours. Solubility is then quantified by total chemiluminescent nitrogen determination (CLND) and values calibrated to molecular weight, number of nitrogens in the compound, and the empirically measured concentration of the sample. These compounds were found to have good aqueous solubility without any noted precipitation and yield different solubility values, suggesting that **42** did not completely transform into **44** in a mostly aqueous solution. While this is not a purely aqueous environment, the compounds are not soluble in 100% water. Unfortunately, the relevance of the stability of the compounds in purely aqueous solutions cannot be investigated, but appears that storing in a solution of DMSO, as is customary for small molecule screens, is more problematic than a mixture of organic and aqueous solvents. Due to compounds exclusively being stored in DMSO,

we found it important to inform readers of the instability that could occur in their compound libraries and repurchased compound stocks for biological testing prior to addition to biological assays. We have expanded our Discussion to include some comments about aqueous solubility and inferred stability. In addition, the Conclusion was updated to omit the grouping of poor organic and aqueous solubility.

For compounds **13** and **37** we describe the literature reported mechanism of reaction with cysteine. We also confirm via mass spectrometry that these compounds covalently interact with several proteins. This is a well characterized mechanism of chemical instability for this chemical series. The reported solubility of **13** and **37**, as determined by nephelometry in phosphate-buffered saline (PBS), demonstrated that these compounds also have good aqueous solubility, at least over a shorter time course.

3. I do not have the expertise to assess aspects of the chemistry in this manuscript, but found other technical aspects to be rigorous. Although the compounds identified are far from ideal, the approach is interesting and my lead to future success by the authors or others. My concerns are that the lack of specificity must be presented in the abstract and other aspects of potential chemical instability of the identified compounds, especially in aqueous solutions, should be addressed.

   Author reply: We are thankful for the reviewers' complimentary comments about the manuscript and the rigor of our experiments. We agree that the specificity of our compounds limits their utility and have included a statement about this lack of specificity in the modified abstract. We have discussed the surprisingly good aqueous solubility of our lead compounds and inferred stability of **42** in a mostly aqueous solution. Part of our message is that these compounds have chemical instabilities, which contribute to their poor selectivity, and your suggested edits have helped us more clearly convey that information, especially in the updated Conclusion. We hope that our compounds help guide future research on SYK or other proteins where similar compounds are uncovered.
