# Supplementary material for: Characterization of covalent inhibitors that disrupt the interaction between the tandem SH2 domains of SYK and FCER1G phospho-ITAM
Source: PLoS One. 2024 Feb 15;19(2):e0293548. doi: 10.1371/journal.pone.0293548 (PMC10868801; doi:10.1371/journal.pone.0293548)
Supplement: Peer review history

# Peer review: Decision Letter 0

**DOI:** 10.1371/journal.pone.0293548.r001

**Authors:** Yanlin Yu

7 Sep 2023

PONE-D-23-24596Characterization of covalent inhibitors that disrupt the interaction between the tandem SH2 domains of SYK and FCER1G phospho-ITAMPLOS ONE

Dear Dr. Axtman,

Thank you for submitting your manuscript to PLOS ONE. After careful consideration, we feel that it has merit but does not fully meet PLOS ONE’s publication criteria as it currently stands. Therefore, we invite you to submit a revised version of the manuscript that addresses the points raised during the review process.

Please submit your revised manuscript by Oct 22 2023 11:59PM. If you will need more time than this to complete your revisions, please reply to this message or contact the journal office at plosone@plos.org. When you're ready to submit your revision, log on to https://www.editorialmanager.com/pone/ and select the 'Submissions Needing Revision' folder to locate your manuscript file.

Please include the following items when submitting your revised manuscript:

- A rebuttal letter that responds to each point raised by the academic editor and reviewer(s). You should upload this letter as a separate file labeled 'Response to Reviewers'.
- A marked-up copy of your manuscript that highlights changes made to the original version. You should upload this as a separate file labeled 'Revised Manuscript with Track Changes'.
- An unmarked version of your revised paper without tracked changes. You should upload this as a separate file labeled 'Manuscript'.

If you would like to make changes to your financial disclosure, please include your updated statement in your cover letter. Guidelines for resubmitting your figure files are available below the reviewer comments at the end of this letter.

If applicable, we recommend that you deposit your laboratory protocols in protocols.io to enhance the reproducibility of your results. Protocols.io assigns your protocol its own identifier (DOI) so that it can be cited independently in the future. For instructions see: https://journals.plos.org/plosone/s/submission-guidelines#loc-laboratory-protocols. Additionally, PLOS ONE offers an option for publishing peer-reviewed Lab Protocol articles, which describe protocols hosted on protocols.io. Read more information on sharing protocols at https://plos.org/protocols?utm_medium=editorial-email&utm_source=authorletters&utm_campaign=protocols.

We look forward to receiving your revised manuscript.

Kind regards,

Yanlin Yu

Academic Editor

PLOS ONE

Journal Requirements:

When submitting your revision, we need you to address these additional requirements.

1. Please ensure that your manuscript meets PLOS ONE's style requirements, including those for file naming. The PLOS ONE style templates can be found at 

https://journals.plos.org/plosone/s/file?id=wjVg/PLOSOne_formatting_sample_main_body.pdf and 

https://journals.plos.org/plosone/s/file?id=ba62/PLOSOne_formatting_sample_title_authors_affiliations.pdf

2. We note that the grant information you provided in the ‘Funding Information’ and ‘Financial Disclosure’ sections do not match. 

When you resubmit, please ensure that you provide the correct grant numbers for the awards you received for your study in the ‘Funding Information’ section.

3. Please expand the acronym “NIA” (as indicated in your financial disclosure) so that it states the name of your funders in full.

This information should be included in your cover letter; we will change the online submission form on your behalf.

4. Thank you for stating the following in the Acknowledgments Section of your manuscript: 

"The authors thank Drs. Allan I. Levey, Lara M. Mangravite, Haian Fu, Opher Gileadi, Aled Edwards, Frank Longo, Gregory W. Carter, Tim M. Willson, and Stephen V. Frye for insightful discussions and suggestions during the development of these resources. The Target Enablement to Accelerate Therapy Development for Alzheimer’s Disease (TREAT-AD) Consortium was established by the National Institute on Aging (NIA). The research reported in this manuscript was led by the Emory-Sage-SGC TREAT-AD center and supported by grant U54AG065187 from the NIA. The Structural Genomics Consortium (SGC) is a registered charity (number 1097737) that receives funds from Bayer AG, Boehringer Ingelheim, the Canada Foundation for Innovation, Eshelman Institute for Innovation, Genentech, Genome Canada through Ontario Genomics Institute [OGI-196], EU/EFPIA/OICR/McGill/KTH/Diamond, Innovative Medicines Initiative 2 Joint Undertaking [EUbOPEN grant 875510], Janssen, Merck KGaA (aka EMD in Canada and USA), Pfizer, the São Paulo Research Foundation-FAPESP, and Takeda. The funders did not play a role in study design, data collection and interpretation, decision to publish, or manuscript preparation."

We note that you have provided funding information that is not currently declared in your Funding Statement. However, funding information should not appear in the Acknowledgments section or other areas of your manuscript. We will only publish funding information present in the Funding Statement section of the online submission form. 

Please remove any funding-related text from the manuscript and let us know how you would like to update your Funding Statement. Currently, your Funding Statement reads as follows: 

"The research reported in this manuscript was led by the Emory-Sage-SGC TREAT-AD center and supported by grant U54AG065187 from the NIA. The Structural Genomics Consortium (SGC) is a registered charity (number 1097737) that receives funds from Bayer AG, Boehringer Ingelheim, the Canada Foundation for Innovation, Eshelman Institute for Innovation, Genentech, Genome Canada through Ontario Genomics Institute [OGI-196], EU/EFPIA/OICR/McGill/KTH/Diamond, Innovative Medicines Initiative 2 Joint Undertaking [EUbOPEN grant 875510], Janssen, Merck KGaA (aka EMD in Canada and USA), Pfizer, the São Paulo Research Foundation-FAPESP, and Takeda. The funders did not play a role in study design, data collection and interpretation, decision to publish, or manuscript preparation."

Please include your amended statements within your cover letter; we will change the online submission form on your behalf.

5. One of the noted authors is a group or consortium [Emory-Sage-SGC TREAT-AD Center ]. In addition to naming the author group, please list the individual authors and affiliations within this group in the acknowledgments section of your manuscript. Please also indicate clearly a lead author for this group along with a contact email address.

6. We note that you have included the phrase “data not shown” in your manuscript. Unfortunately, this does not meet our data sharing requirements. PLOS does not permit references to inaccessible data. We require that authors provide all relevant data within the paper, Supporting Information files, or in an acceptable, public repository. Please add a citation to support this phrase or upload the data that corresponds with these findings to a stable repository (such as Figshare or Dryad) and provide and URLs, DOIs, or accession numbers that may be used to access these data. Or, if the data are not a core part of the research being presented in your study, we ask that you remove the phrase that refers to these data.

7. PLOS ONE now requires that authors provide the original uncropped and unadjusted images underlying all blot or gel results reported in a submission’s figures or Supporting Information files. This policy and the journal’s other requirements for blot/gel reporting and figure preparation are described in detail at https://journals.plos.org/plosone/s/figures#loc-blot-and-gel-reporting-requirements and https://journals.plos.org/plosone/s/figures#loc-preparing-figures-from-image-files. When you submit your revised manuscript, please ensure that your figures adhere fully to these guidelines and provide the original underlying images for all blot or gel data reported in your submission. See the following link for instructions on providing the original image data: https://journals.plos.org/plosone/s/figures#loc-original-images-for-blots-and-gels. 

  

In your cover letter, please note whether your blot/gel image data are in Supporting Information or posted at a public data repository, provide the repository URL if relevant, and provide specific details as to which raw blot/gel images, if any, are not available. Email us at plosone@plos.org if you have any questions.

8. Please review your reference list to ensure that it is complete and correct. If you have cited papers that have been retracted, please include the rationale for doing so in the manuscript text, or remove these references and replace them with relevant current references. Any changes to the reference list should be mentioned in the rebuttal letter that accompanies your revised manuscript. If you need to cite a retracted article, indicate the article’s retracted status in the References list and also include a citation and full reference for the retraction notice.

Additional Editor Comments:

PONE-D-23-24596

Characterization of covalent inhibitors that disrupt the interaction between the tandem SH2 domains of SYK and FCER1G phospho-ITAM

Dear Dr. ,

Thank you for submitting your manuscript to PLOS ONE. After careful consideration, the referees and editors expressed interest in the study, but they also have a number of criticisms and suggestions. We would be interested in considering a revised version of the manuscript that addresses these concerns in detail.

However, please note that the revised manuscript may have to be re-reviewed, which might result in additional delays to publication while the original reviewers assess the resubmission. If the reviewers give a low rating, we will not be able to accept the manuscript for publication.

The review comments are enclosed at the end of this email.

Sincerely,

Yanlin Yu, Ph.D.

Academic Editor of PLOS One

Reviewers' comments:

Bashore et al. have screened a large (>138k) chemical library for compounds that disrupt the interaction between the SH2 domains on SYK protein tyrosine kinase and the phosphorylated ITAM domains of FCER1G. Development of such compounds has therapeutic potential in treating Alzheimer’s and other diseases. They identified several compounds that efficiently disrupted the interaction when used at micromolar concentrations, and many of these effective compounds covalently attached to cysteines and other residues in the SH2 domain. The authors provide technically rigorous evidence to support their conclusions through use of a variety of state-of-the-art techniques, including FRET, bio-layer interferometry, mass spectrometry, and orthogonal-based GST pull-down assays. While I do not have any criticism of the technical aspects of the work, the authors should address two concerns:

1. The primary concern for future development of these compounds as potential therapeutics is their lack of specificity to the cysteine residues in SH2 domains of Syk, since they can readily form covalent interactions with available cysteines on other proteins. Nonetheless, the approach is still interesting and future modifications may improve specificity. This lack of specificity is well documented by the authors in the Results, Discussion, and Conclusions sections, but this fact should also be prominently stated in the Abstract.

2. While the authors documented the instability of the inactive compound 42 to the active compound 44 in DMSO, they should also comment in Discussion on any other observed or potential instabilities of the active compounds that they have identified when suspended in aqueous solutions, which would be more biologically relevant.

I do not have the expertise to assess aspects of the chemistry in this manuscript, but found other technical aspects to be rigorous. Although the compounds identified are far from ideal, the approach is interesting and my lead to future success by the authors or others. My concerns are that the lack of specificity must be presented in the abstract and other aspects of potential chemical instability of the identified compounds, especially in aqueous solutions, should be addressed.

[Note: HTML markup is below. Please do not edit.]

Reviewers' comments:

Reviewer's Responses to Questions

**Comments to the Author**

1. Is the manuscript technically sound, and do the data support the conclusions?

The manuscript must describe a technically sound piece of scientific research with data that supports the conclusions. Experiments must have been conducted rigorously, with appropriate controls, replication, and sample sizes. The conclusions must be drawn appropriately based on the data presented. 

Reviewer #1: Yes

**********

2. Has the statistical analysis been performed appropriately and rigorously? 

Reviewer #1: Yes

**********

3. Have the authors made all data underlying the findings in their manuscript fully available?

The PLOS Data policy requires authors to make all data underlying the findings described in their manuscript fully available without restriction, with rare exception (please refer to the Data Availability Statement in the manuscript PDF file). The data should be provided as part of the manuscript or its supporting information, or deposited to a public repository. For example, in addition to summary statistics, the data points behind means, medians and variance measures should be available. If there are restrictions on publicly sharing data—e.g. participant privacy or use of data from a third party—those must be specified.

Reviewer #1: Yes

**********

4. Is the manuscript presented in an intelligible fashion and written in standard English?

PLOS ONE does not copyedit accepted manuscripts, so the language in submitted articles must be clear, correct, and unambiguous. Any typographical or grammatical errors should be corrected at revision, so please note any specific errors here.

Reviewer #1: Yes

**********

5. Review Comments to the Author

Please use the space provided to explain your answers to the questions above. You may also include additional comments for the author, including concerns about dual publication, research ethics, or publication ethics. (Please upload your review as an attachment if it exceeds 20,000 characters)

Reviewer #1: Bashore et al. have screened a large (>138k) chemical library for compounds that disrupt the interaction between the SH2 domains on SYK protein tyrosine kinase and the phosphorylated ITAM domains of FCER1G. Development of such compounds has therapeutic potential in treating Alzheimer’s and other diseases. They identified several compounds that efficiently disrupted the interaction when used at micromolar concentrations, and many of these effective compounds covalently attached to cysteines and other residues in the SH2 domain. The authors provide technically rigorous evidence to support their conclusions through use of a variety of state-of-the-art techniques, including FRET, bio-layer interferometry, mass spectrometry, and orthogonal-based GST pull-down assays. While I do not have any criticism of the technical aspects of the work, the authors should address two concerns:

1. The primary concern for future development of these compounds as potential therapeutics is their lack of specificity to the cysteine residues in SH2 domains of Syk, since they can readily form covalent interactions with available cysteines on other proteins. Nonetheless, the approach is still interesting and future modifications may improve specificity. This lack of specificity is well documented by the authors in the Results, Discussion, and Conclusions sections, but this fact should also be prominently stated in the Abstract.

2. While the authors documented the instability of the inactive compound 42 to the active compound 44 in DMSO, they should also comment in Discussion on any other observed or potential instabilities of the active compounds that they have identified when suspended in aqueous solutions, which would be more biologically relevant.

**********

6. PLOS authors have the option to publish the peer review history of their article (what does this mean?). If published, this will include your full peer review and any attached files.

If you choose “no”, your identity will remain anonymous but your review may still be made public.

**Do you want your identity to be public for this peer review?** For information about this choice, including consent withdrawal, please see our Privacy Policy.

Reviewer #1: No

**********

[NOTE: If reviewer comments were submitted as an attachment file, they will be attached to this email and accessible via the submission site. Please log into your account, locate the manuscript record, and check for the action link "View Attachments". If this link does not appear, there are no attachment files.]

While revising your submission, please upload your figure files to the Preflight Analysis and Conversion Engine (PACE) digital diagnostic tool, https://pacev2.apexcovantage.com/. PACE helps ensure that figures meet PLOS requirements. To use PACE, you must first register as a user. Registration is free. Then, login and navigate to the UPLOAD tab, where you will find detailed instructions on how to use the tool. If you encounter any issues or have any questions when using PACE, please email PLOS at figures@plos.org. Please note that Supporting Information files do not need this step.

# Author comment: Author response to Decision Letter 0

**DOI:** 10.1371/journal.pone.0293548.r002

22 Sep 2023

All edits requested by the reviewer have been addressed and are described in the uploaded Response to Reviewers document.

Attachment Submitted filename: Response to Reviewers.pdf Click here for additional data file.

# Peer review: Decision Letter 1

**DOI:** 10.1371/journal.pone.0293548.r003

**Authors:** Yanlin Yu

16 Oct 2023

Characterization of covalent inhibitors that disrupt the interaction between the tandem SH2 domains of SYK and FCER1G phospho-ITAM

PONE-D-23-24596R1

Dear Dr. Axtman,

We’re pleased to inform you that your manuscript has been judged scientifically suitable for publication and will be formally accepted for publication once it meets all outstanding technical requirements.

Within one week, you’ll receive an e-mail detailing the required amendments. When these have been addressed, you’ll receive a formal acceptance letter and your manuscript will be scheduled for publication.

An invoice for payment will follow shortly after the formal acceptance. To ensure an efficient process, please log into Editorial Manager at http://www.editorialmanager.com/pone/, click the 'Update My Information' link at the top of the page, and double check that your user information is up-to-date. If you have any billing related questions, please contact our Author Billing department directly at authorbilling@plos.org.

If your institution or institutions have a press office, please notify them about your upcoming paper to help maximize its impact. If they’ll be preparing press materials, please inform our press team as soon as possible -- no later than 48 hours after receiving the formal acceptance. Your manuscript will remain under strict press embargo until 2 pm Eastern Time on the date of publication. For more information, please contact onepress@plos.org.

Kind regards,

Yanlin Yu

Academic Editor

PLOS ONE

Additional Editor Comments (optional):

Reviewers' comments:

Reviewer's Responses to Questions

**Comments to the Author**

1. If the authors have adequately addressed your comments raised in a previous round of review and you feel that this manuscript is now acceptable for publication, you may indicate that here to bypass the “Comments to the Author” section, enter your conflict of interest statement in the “Confidential to Editor” section, and submit your "Accept" recommendation.

Reviewer #1: All comments have been addressed

**********

2. Is the manuscript technically sound, and do the data support the conclusions?

The manuscript must describe a technically sound piece of scientific research with data that supports the conclusions. Experiments must have been conducted rigorously, with appropriate controls, replication, and sample sizes. The conclusions must be drawn appropriately based on the data presented. 

Reviewer #1: Yes

**********

3. Has the statistical analysis been performed appropriately and rigorously? 

Reviewer #1: Yes

**********

4. Have the authors made all data underlying the findings in their manuscript fully available?

The PLOS Data policy requires authors to make all data underlying the findings described in their manuscript fully available without restriction, with rare exception (please refer to the Data Availability Statement in the manuscript PDF file). The data should be provided as part of the manuscript or its supporting information, or deposited to a public repository. For example, in addition to summary statistics, the data points behind means, medians and variance measures should be available. If there are restrictions on publicly sharing data—e.g. participant privacy or use of data from a third party—those must be specified.

Reviewer #1: Yes

**********

5. Is the manuscript presented in an intelligible fashion and written in standard English?

PLOS ONE does not copyedit accepted manuscripts, so the language in submitted articles must be clear, correct, and unambiguous. Any typographical or grammatical errors should be corrected at revision, so please note any specific errors here.

Reviewer #1: Yes

**********

6. Review Comments to the Author

Please use the space provided to explain your answers to the questions above. You may also include additional comments for the author, including concerns about dual publication, research ethics, or publication ethics. (Please upload your review as an attachment if it exceeds 20,000 characters)

Reviewer #1: The authors have addressed my previous minor concerns. The manuscript is technically sound and conclusions are well supported by the data provided. Statistics are adequate.

**********

7. PLOS authors have the option to publish the peer review history of their article (what does this mean?). If published, this will include your full peer review and any attached files.

If you choose “no”, your identity will remain anonymous but your review may still be made public.

**Do you want your identity to be public for this peer review?** For information about this choice, including consent withdrawal, please see our Privacy Policy.

Reviewer #1: No

**********

# Editor report: Acceptance letter

**DOI:** 10.1371/journal.pone.0293548.r004

**Authors:** Yanlin Yu

19 Oct 2023

PONE-D-23-24596R1 

Characterization of covalent inhibitors that disrupt the interaction between the tandem SH2 domains of SYK and FCER1G phospho-ITAM 

Dear Dr. Axtman:

I'm pleased to inform you that your manuscript has been deemed suitable for publication in PLOS ONE. Congratulations! Your manuscript is now with our production department. 

If your institution or institutions have a press office, please let them know about your upcoming paper now to help maximize its impact. If they'll be preparing press materials, please inform our press team within the next 48 hours. Your manuscript will remain under strict press embargo until 2 pm Eastern Time on the date of publication. For more information please contact onepress@plos.org.

If we can help with anything else, please email us at plosone@plos.org. 

Thank you for submitting your work to PLOS ONE and supporting open access. 

Kind regards, 

PLOS ONE Editorial Office Staff

on behalf of

Dr. Yanlin Yu 

Academic Editor

PLOS ONE
